# Heart Failure Epidemiology: European Perspective

**DOI:** 10.2174/1573403X11309020005

**Published:** 2013-05

**Authors:** K Guha, T McDonagh

**Affiliations:** 1 Royal Brompton Hospital, Chelsea, London, UK; 2 King’s College Hospital, Denmark Hill, London, UK

**Keywords:** Epidemiology, heart failure, cardiology, LVSD, MI.

## Abstract

Heart failure poses an increasing problem for global healthcare systems. The epidemiological data which has been accrued over the last thirty years has predominantly been accumulated from experience within North America and Europe.

Initial large cohort, prospective longitudinal studies produced the first publications; however latterly the focus has shifted onto epidemiological data governing hospitalisation and mortality. The emphasis behind this shift has been the resource implications with regards to repetitive, costly and prolonged hospitalisation. The European experience in heart failure, though similar to North America has recently demonstrated differences in hospitalisation which may underlie the differences between healthcare system configuration.

Heart failure however remains an increasing global problem and the endpoint of a variety of cardiovascular diseases. Allied with the fact of increasingly elderly populations and prior data demonstrating a steep rise in prevalent cases within more elderly populations, it is likely that the increasing burden of disease will continue to pose challenges for modern healthcare.

Despite the predicted increase in the number of patients affected by heart failure, over the last thirty years, a clear management algorithm has evolved for the use of pharmacotherapies (neuro-hormonal antagonists), device based therapies (Implantable Cardioverting Defibrillator (ICD) and Cardiac Resynchronisation Therapy (CRT)) and mechanical therapies including left ventricular assist devices and cardiac transplantation. Though the management of such patients has been clearly delineated in national and international guidelines, the underuse of all available and appropriate therapies remains a significant problem.

When comparing various epidemiological studies from different settings and timepoints, it should be remembered that rates of prevalence and incidence may vary depending upon the definition used, methods of accumulating information (with the possibility of bias) and the chosen cut point of defining left ventricular systolic dysfunction (LVSD).

## DEFINITION OF HEART FAILURE

Heart failure is a collection of signs, symptoms and pathophysiology. The definition of heart failure has evolved, with the current European Society of Cardiology saying that it is ‘Heart failure is a clinical syndrome in which patients have typical symptoms and signs resulting from an abnormality of cardiac structure and function [1].

**Fig. (1):**
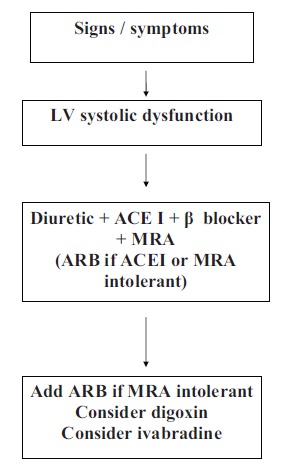
Demonstrates Treatment Algorithm for Left Ventricular Systolic Dysfunction. ACEI = Ace Inhibitor, BB = Beta Blocker, MRA = Mineralocorticoid Receptor, ARB= Angiotensin II Receptor Antagonist

Many historical methods of sub classification have been proposed for dividing patients into cohorts including forward and backward, high and low output states. In the 1990s, the emphasis was on systolic dysfunction as the main cardiac abnormality underlying heart failure. If proven via cardiac imaging, then the patient is a candidate for the management algorithm demonstrated in (Fig. **1**). However, this is not the entire problem. There is a growing cohort of patients who exhibit signs, symptoms and some aspects of the pathophysiology of heart failure (evidence of raised left ventricular filling pressures on echocardiography) who have preserved left ventricular systolic function (Heart Failure with Preserved Ejection Fraction – HF-PEF). This group of patients has a similar mortality to populations with LVSD, yet remains relatively poorly within compared to systolic dysfunction. Further data is needed within such groups to clarify appropriate management and identification of such individuals. 

The use of natriuretic peptide assays has also led to the discovery that not all LVSD is symptomatic. Increased awareness and selective studies have demonstrated that a significant cohort of patients exist with asymptomatic marked LVSD. The prognosis for such individuals is analogous to those with symptoms. Due to the organisation of healthcare systems and current economic climate, most cases detected reflect symptomatic disease. Targeted screening using a combination of neurohormonal peptides and cardiac imaging such as echocardiography ensure that LVSD is detected in high risk populations (e.g. prior myocardial infarction (MI)).

## INCIDENCE

Whereas in the US, longitudinal cohorts were created to study the effects of cardiovascular disease from the 1950s onwards, which led to the publications from Framingham and Olmsted county cohorts. In Europe the ‘Men born in 1913 study’ was the first to report [2-4]. Within this study 973 participants were randomly chosen from the electoral roll in Gothenburg, Sweden. All were aged 50 at enrollment which occurred in 1963, hence the pseudonym. They were followed up annually, and the results published in 1989. Using a clinical scoring system, predominately based on symptoms, the group demonstrated a rise in incidence in cases in HF with age. The incidence rose from 2.1 per 1000 at age 50 to 8.2 per 1000 aged 67. The pattern of increasing incidence and prevalence of HF with age was to become a familiar one. 

The Hillingdon heart failure study group created a study in the 1990s, to ascertain incidence [5]. Using the west London district of Hillingdon, all incident cases of HF within the region were identified either via emergency admissions or a specialist dedicated clinic. The district covered 151,000 patients covered by 82 general practices. 220 new cases were identified during the study period. Using chest radiography, electrocardiograms and echocardiograms, a panel of three cardiologists was able to make a consensus decision as to diagnosis. The incidence rose within the cohort from 0.02/1000 per year in the 25-34 age group to 11.6/1000 in those aged over 85 years. The median age at presentation was 76 years. The findings from the Hillingdon HF study were consistent with another study based within primary care. Using the General Practice Research Database in the United Kingdom, 696,884 patients above the age of 45 years were identified [6]. Using a grading system based on medical documentation and prescription habits, patients were categorised into definitive, possible and non HF groups. The incidence of definitive HF was 9.3/1000 per year with a mean age of the population with definitive heart failure being 77 years of age. Scottish data also corroborates the initial findings, with a dataset from the Scottish Continuous Morbidity recording system demonstrating a rise in incidence of 2 per 1000 per year to 22 per1000 per year in men aged above 85yrs old [7]. 

## PREVALENCE

The prevalence of heart failure can be divided between those studies which are focused on community and those which are specific to certain populations. One of the initial population based studies was based in North-West London, where 30, 204 primary care case records were reviewed [8]. This produced a prevalence of HF cases of 0.6 per 1000 for those aged below 65 years rising to 28 per 1000 in those aged above 65. One problem which recurs throughout most primary care studies, is the diagnostic method used to identify cases. A Swedish study using primary care centres chosen at random throughout Sweden, demonstrated 30% of HF patients had been diagnosed on the basis of symptoms and signs and chest radiography. Within this cohort there was an underutilisation of HF evidence based therapies [9].

Another study based in Utrecht, Netherlands found that patients who were under a cardiologist were likely to be younger, with an aetiology of ischaemic heart disease [10]. This illustrates a recurrent issue within HF epidemiology. There is a large pool of individuals within the community with HF, who are elderly, frail and have multiple comorbidities. However their access to cardiology, cardiac services and evidence based therapy is poor. This leads to problems with frequent prolonged hospitalisation and ultimately higher rates of morbidity and mortality. This carries significant risk for the individual but also financial consequences for publicly funded economies. A recent Scottish study is also supportive of such a viewpoint. Using the Scottish Continuous Morbidity reporting scheme, and accessing 57 general practices across Scotland with HF Read codes for 307, 741 patients, McMurray *et al.* demonstrated that the prevalence of HF within the general Scottish population rose from 7.1 per 1000 in the below 65 years of age, rising to 90.1 per 1000 in the above 85 year old population [7].

## POPULATION BASED STUDIES USING SCREENING TECHNIQUES (ECHOCARDIOGRAPHY)

The initial study which described the population prevalence was the MONICA study set in North Glasgow [11]. The study consisted of a randomised sample of 2000 individuals (male and female) aged between 25-74 years of age, basic transthoracic echocardiography was used to find index cases. The chosen cut point was a left ventricular ejection fraction (LVEF) of less than 30 percent. 2.9 % of the cohort had left ventricular systolic dysfunction. Approximately half the patients were taking a diuretic or were breathless. (1.5%) and the remainder (1.4%) had asymptomatic significant LVSD. The prevalence rose with age and was higher in men versus women. 

Similar findings have been documented within other studies. The ECHOES Study based within the Midlands, United Kingdom documented a prevalence of 1.8-3.5% with again 50% of patients having asymptomatic LVSD [12]. The cross sectional EPICA study based in Portugal, which covered 5434 participants, demonstrated a steep rise in prevalence from 1.36% in the 25-49 year old age group to 16.14% in the above 80 year old age group [13]. The overall prevalence due to LVSD was 1.3%, with 1.7% being those with preserved LVEF. 

Due to the relationship with age, some studies have exclusively studied more elderly cohorts. The Helsinki Ageing Study based in Finland, evaluated 501 subjects aged between 75-86 years of age [14]. The overall clinical prevalence of HF was 8.2%, 2.3% had LVSD and 9% ALVSD. In Rotterdam, Netherlands an analysis of 2267 individuals, aged 55-95 years of age had fractional shortening less than 25% (5.5% male, 2.2% female) and 2.2% were asymptomatic [15]. Another study located within Poole, Dorset, United Kingdom showed with a cohort of 817 participants, aged between 70-84 years of age, 7.5% had LVSD with an increased rate observed within men of 12.5% [16]. 

With the increase in incidence and prevalence with age, some groups have even examined prevalence rates within the very elderly. In the BELFRAIL study performed in Belgium, 567 individuals over the age of 80 were identified. Using domiciliary based echocardiography 5.8% of the cohort had LVSD, with preserved LV systolic function HF being 3.1% [17]. 

## HEART FAILURE WITH PRESERVED EJECTION FRACTION (HF-PEF)

The studies described above were predominately tailored towards the detection of LVSD. This is due to the large evidence base for recommended pharmacotherapies and device based therapies for this population. However most of the prevalence studies also reveal a similar rate of HF-PEF i.e. heart failure symptoms and signs with preserved LV systolic function. A review of prevalence of the condition suggests prevalence ranging between 1.5 -4.8% depending on the study [18]. 

Within the ECHOES study 1.1% had HF and a LVEF >50%, whereas in the Helsinki study 72% of all the cases of HF had preserved LV systolic function [12, 14]. The EPICA study demonstrated a prevalence across all age groups of 1.7% with preserved LV systolic function [13]. 

## HEART FAILURE HOSPITALISATION

Heart failure is responsible in most Westernised economies for 1-2% of all healthcare expenditure [19]. The bulk of these costs are driven by frequent, prolonged and repeat hospitalisations. Data was collected in 1990s, from Sweden, Scotland and the Netherlands [20-22]. In Scotland, 0.2% of the general population were hospitalised with HF per annum, with 5% of all medical admissions being due to HF. However despite the initial data, HF hospitalisation rates may have peaked within the 1990s. More recent Scottish data is suggestive that HF hospitalisations peaked in the 1990s and subsequently declined by 2003 [23]. Similar findings have also been described from Dutch data [24]. 

## HEART FAILURE PROGNOSIS

The prognosis for HF patients has remained alarmingly poor over the last twenty years. Differing mortality rates have been described depending on whether the study was community or hospital based. Mortality rates within the ECHOES study were 47% over five years for those with LVSD and 38% for those with HF-NEF [25]. The MONICA study also demonstrated that ALVSD is an important clinical entity, 21% of the cohort were dead at 4 years [26].

The mortality amongst patients in the community remains high, but the most lethal place for heart failure patients remains those hospitalised. Several studies and registries have documented high rates of mortality. The Euroheart II registry published in 2006 showed a 6.6% mortality rate in –hospital [27]. The National Health Service audit covering England and Wales illustrated an in hospital mortality of 15 % [28]. More recently the national audit data for England and Wales demonstrates little improvement in terms of disease management with a rate of in patient mortality of 12% and a 1 year mortality rate of 26% below the age of 75 and 56% for those aged above 75 [29]. 

The issues of case detection, management and accessibility to in and out patient cardiac investigations are cyclical and problematic. Despite the wealth of evidence which spawned several national and international guidelines, it is clear that several hospitalised patients with HF remain on sub optimal medical therapy and prone to repeat admission [1]. Also with the bulk of the disease falling within more elderly populations it is also relevant that more elderly groups are not commenced on evidence based therapies and hence experience higher rates of morbidity and mortality. 

## AETIOLOGY

Initial epidemiological studies revealed the leading cause of LVSD to be hypertension [2]. However over the last four decades this has gradually changed to become ischaemic heart disease (IHD) [27]. The geographical variance of IHD rates across Europe is exposed by the trial data. The rate of IHD as an aetiology in the MONICA study was 95%, with 53% in the ECHOES study and in the Helsinki Study it was 54% [12, 30]. Conversely within the EPICA study, the rate of IHD was 39% [13]. This may reflect the susceptibility of certain countries and populations to IHD. 

Populations with high rates of IHD normally also have high rates of co-morbidities such as hypertension, with an example being a rate of 68% of hypertension in the MONICA study. 

The majority of heart failure epidemiology has been collected from Western Europe, with notable pan European data predominately coming from European Society of Cardiology initiatives such as the EuroHeart II study [27].

The Bromley Heart Failure study located within Bromley, South-East London was devised to investigate the aetiology of incident heart failure [31]. Using a similar methodical system as previously, the authors demonstrated that out 332 patients were identified [32]. 136 of these 332 cases were under the age of 75 years of age, with 99 (73%) of these proceeding to coronary angiography. Ultimately, 52% of the cohort below the age of 75 years was attributable to IHD. 

However European populations are seeing a large scale influx of immigration with descendants of first generation immigrants settling within European countries. This raises the possibility of similar disease states such as IHD but a different disease trajectory. South Asians have a seemingly higher rate of diabetes, hypertension and tobacco usage. This superimposed upon a genetic predisposition means that they may endure accelerated coronary artery disease with resulting LVSD. Data from Leicester, United Kingdom is supportive of such findings [33]. Rheumatic heart disease though non existent within native European populations, is endemic in Sub-Saharan Africa and South East Asia [34]. With widespread immigration and travel from such areas, this may represent a significant aetiology within certain patient groups. 

## CONCLUSION

Heart failure remains a pertinent problem across Europe. It is responsible for large economic costs, frequent hospitalisation and high levels of mortality. Much epidemiological work has been done across Europe to illustrate prevalence with the acknowledgement that increasing numbers of elderly patients are likely to cause significant problems over the next few decades. Large numbers of particularly elderly patients remain undiagnosed and also on sub-optimal therapies. The problem is likely to become hyper acute over the next two decades with increased accessibility to revascularization and survival following myocardial infarction. The combination of increasingly elderly populations and IHD management will ensure higher prevalence of LVSD amongst most European countries. 

Another important point which is relevant to European populations is the increase in immigration, from different areas of the world with different disease entities. These groups will also undergo an epidemiological transition within the host country and may be subject to conventional risk factors for HF progression e.g. IHD. The disease processes also may behave differently within such individuals. However little is know about these cohorts and further work needs to be carried out in such areas. 

Ultimately similar to other global regions, the real challenge facing European healthcare systems is how to cope with the rise in prevalence and the consequent care at primary, secondary and tertiary care levels. All of this becomes even more relevant with the current global economic backdrop and financial limitations. A large proportion of European citizens are transitioning to becoming elderly or very elderly and will ask further questions of existing systems and models of heart failure care. 

## ABBREVIATIONS

ICD: = Implantable Cardioverting Defibrillator
CRT: = Cardiac Resynchronisation Therapy
LVSD: = Left Ventricular Systolic Dysfunction
HF –PEF: = Heart Failure with Preserved Ejection Fraction
MI: = Myocardial Infarction
HF: = Heart Failure
MONICA: = Multinational Monitoring of Trends and Determinants in Cardiovascular Disease (World Health Organisation)
ECHOES: = Echocardiographic Heart of England Screening
LVEF: = Left Ventricular Ejection Fraction
ALVSD: = Asymptomatic Left Ventricular Systolic Dysfunction
IHD: = Ischaemic Heart Disease
